# Gut microbiome-based noninvasive diagnostic model to predict acute coronary syndromes

**DOI:** 10.3389/fcimb.2023.1305375

**Published:** 2024-01-11

**Authors:** Jincheng Wang, Zhao Hu, Qiuyue Xu, Yunke Shi, Xingyu Cao, Yiming Ma, Mingqiang Wang, Chaoyue Zhang, Xiang Luo, Fanru Lin, Xianbin Li, Yong Duan, Hongyan Cai

**Affiliations:** ^1^ Department of Cardiology, the First Affiliated Hospital of Kunming Medical University, Kunming, China; ^2^ Department of Geriatric Cardiology, the First Affiliated Hospital of Kunming Medical University, Kunming, China; ^3^ Department of Clinical Laboratory, The First Affiliated Hospital of Kunming Medical University, Yunnan Key Laboratory of Laboratory Medicine, Yunnan Province Clinical Research Center for Laboratory Medicine, Kunming, China

**Keywords:** acute coronary syndrome, gut microbiome, diagnostic model, nomogram, 16SrRNA

## Abstract

**Background:**

Previous studies have shown that alterations in the gut microbiota are closely associated with Acute Coronary Syndrome (ACS) development. However, the value of gut microbiota for early diagnosis of ACS remains understudied.

**Methods:**

We recruited 66 volunteers, including 29 patients with a first diagnosis of ACS and 37 healthy volunteers during the same period, collected their fecal samples, and sequenced the V4 region of the 16S rRNA gene. Functional prediction of the microbiota was performed using PICRUSt2. Subsequently, we constructed a nomogram and corresponding webpage based on microbial markers to assist in the diagnosis of ACS. The diagnostic performance and usefulness of the model were analyzed using boostrap internal validation, calibration curves, and decision curve analysis (DCA).

**Results:**

Compared to that of healthy controls, the diversity and composition of microbial community of patients with ACS was markedly abnormal. Potentially pathogenic genera such as *Streptococcus* and *Acinetobacter* were significantly increased in the ACS group, whereas certain SCFA-producing genera such as *Blautia* and *Agathobacter* were depleted. In addition, in the correlation analysis with clinical indicators, the microbiota was observed to be associated with the level of inflammation and severity of coronary atherosclerosis. Finally, a diagnostic model for ACS based on gut microbiota and clinical variables was developed with an area under the receiver operating characteristic (ROC) curve (AUC) of 0.963 (95% CI: 0.925–1) and an AUC value of 0.948 (95% CI: 0.549–0.641) for bootstrap internal validation. The calibration curves of the model show good consistency between the actual and predicted probabilities. The DCA showed that the model had a high net clinical benefit for clinical applications.

**Conclusion:**

Our study is the first to characterize the composition and function of the gut microbiota in patients with ACS and healthy populations in Southwest China and demonstrates the potential effect of the microbiota as a non-invasive marker for the early diagnosis of ACS.

## Introduction

Acute coronary syndromes (ACS), including ST-segment elevation myocardial infarction (STEMI), non-ST-segment elevation myocardial infarction (NSTEMI), and unstable angina (UA), are leading causes of morbidity and mortality worldwide (Bergmark et al., 2022). Its major pathological mechanism is the rupture or erosion of unstable atherosclerotic plaques, leading to myocardial ischaemia and thrombosis, which is characterized by sudden onset and rapid progression, and may lead to malignant and life-threatening lesions at any time if left untreated(Bhatt et al., 2022). Currently, ACS diagnosis relies on clinical symptoms, electrocardiographic dynamics, and alterations in myocardial necrosis markers such as myoglobin, creatine kinase isoenzyme MB (CK-MB), cardiac troponin T (cTnT), and cardiac troponin I (cTnI) (Byrne et al., 2023). However, one of the main problems in the clinical diagnosis ACS is the late onset of disease symptoms or only atypical symptoms, which may lead to a delay in consultation and miss the optimal time to save the patient’s life(Brieger et al., 2004). In addition, myocardial necrosis markers are not released from the myocardium until after myocardial ischaemia and necrosis, and are not elevated in unstable angina or in the early stages of acute myocardial infarction, making it impossible to diagnose early ischaemia(Mair, 1997; Braunwald, 2012). Therefore, the identification of novel biomarkers for the early diagnosis of ACS is an emerging priority, as it may facilitate the timely receipt of appropriate treatment and reduce the mortality and disability of patients(Katus et al., 2017).

Recently, a growing body of evidence has shown that gut microbiota is closely related to the pathogenesis of cardiovascular diseases, particularly coronary artery disease(Koeth et al., 2013; Jie et al., 2017; Zhu et al., 2018; Liu et al., 2019), hypertension(Yang et al., 2015), and heart failure(Pasini et al., 2016). This interaction between the gut and the heart is known as the “gut-heart axis”(Du et al., 2020; Troseid et al., 2020). On the one hand, Dysbiosis of the gut microbiota contributes to the progression of cardiovascular diseases by manipulating the host immune response and exacerbating the inflammatory response(Chistiakov et al., 2015; van den Munckhof et al., 2018). On the other hand, the microbial community in the gut can produce various metabolites, including trimethylamine oxides, bile acids, and short-chain fatty acids, which enter the systemic circulation and affect the host’s lipid metabolism, bile acid metabolism, and energy metabolism(Zhu et al., 2016; Fatkhullina et al., 2018; Haghikia et al., 2022; Tousoulis et al., 2022). Numerous studies have demonstrated the presence of gut microbiota dysbiosis in patients with coronary artery disease, accompanied by changes in the structure, composition and function of the microbiota(Jie et al., 2017; Zhu et al., 2018). Furthermore, a diagnostic model based on gut microbiota and clinical features was developed to improve the diagnostic performance of CAD(Zheng et al., 2020). Recently, the composition and function of the gut microbiota were shown to vary in patients with different subtypes of coronary artery disease. In particular, the microbiota profile of patients with ACS is significantly different from that of patients with stable coronary artery disease(Liu et al., 2019; Khan et al., 2022; Dong et al., 2023). However, no study has established a gut microbiome-based diagnostic model for ACS.

To address these questions, we investigated the characteristics and differences in gut microbiota composition between patients with ACS and healthy populations by 16S rRNA gene sequencing and explored the effectiveness of gut microbiota as a tool for early diagnosis of ACS.

## Materials and method

### Study population

This was a single-center cross-sectional study. We consecutively recruited 29 patients with newly diagnosed ACS, including those with ST-segment elevation myocardial infarction (STEMI), non-ST-segment elevation myocardial infarction (NSTEMI), and unstable angina (UA), January 2022–June 2022 at the First Affiliated Hospital of Kunming Medical University. The diagnosis of ACS was based on a combination of clinical symptoms, electrocardiograms, myocardial enzymes, and coronary angiography and the detailed diagnostic criteria were based on the ESC guidelines(Byrne et al., 2023). The control group comprised 37 asymptomatic healthy volunteers who underwent routine physical examination at the First Affiliated Hospital of Kunming Medical University during the same period. All participants were long-term local residents, aged between 18 and 80 years, and volunteered to provide a complete medical history, clinical examination parameters, and stool samples. We excluded patients with previous coronary artery disease, heart failure, structural heart disease, gastrointestinal disease (including peptic ulcer, acute gastroenteritis, inflammatory bowel disease, etc.), severe liver or kidney disease, autoimmune disease, malignant tumours, antibiotic or probiotic use in the past three months, and those with a history of prolonged diarrhoea or constipation. The study protocol was approved by the Ethics Committee of the First Affiliated Hospital of Kunming Medical University and all patients provided written informed consent to participate in the study. All procedures were performed in accordance with the ethical standards of the Declaration of Helsinki and its subsequent revisions.

### Clinical data and sample collection

Clinical data were collected from all participants, including demographic characteristics such as age, sex, height, weight, smoking and drinking habits, and medical history. All patients underwent coronary angiography, the results of which were independently confirmed by two professional cardiologists, and the severity of coronary atherosclerosis was assessed using the Gensini score(Gensini, 1983). Five milliliters of fasting peripheral venous blood were collected from each participant on the morning of the day following admission, and routine blood tests, liver function, renal function, and lipid analyses were performed. All participants were asked to collect stool samples within the next day of admission and were trained in sample collection. Each subject was provided with a sterile disposable tray and sterile stool sampler with a spoon for stool sample collection by researchers beforehand. All participants were asked to empty their urine, wash their hands, and wear disposable gloves prior to stool collection. The subjects’ stools were collected in sterile disposable trays and the middle portion of the stool was collected using a sterile stool sampler with a spoon. Each subject’s stool sample was then divided equally into five portions of 200 mg each and transported immediately to the laboratory for freezing at -80°C.

### DNA extraction and 16S rRNA gene V4 region sequencing

The fecal bacterial DNA was extracted using cetyltrimethylammonium bromide (CTAB) method. The DNA concentration and purity were monitored on a 1% agarose gel. According to the concentration, DNA was diluted to 1ng/µL using sterile water. The V4 region of the 16S rRNA gene was amplified by polymerase chain reaction (PCR) using the extracted DNA as template. The sequence of the forwarding primers used was 515F(5’-GTGCCAGCMGCCGCGGTAA-3’) and the reverse primer used sequence was 806R (5’-GGACTACHVGGGTWTCTAAT-3’). Sequencing libraries were generated using the TruSeq^®^ DNA PCR-Free Sample Preparation Kit (Illumina, USA) and index codes were added according to the manufacturer’s recommendations. Library quality was assessed using a Qubit@ 2.0 Fluorometer (Thermo Scientific) and an Agilent Bioanalyzer 2100 system. Finally, the validated libraries were sequenced using an Illumina NovaSeq 6000 (NovoGene, Beijing, China), generating 250 bp paired-end reads according to the manufacturer’s instructions.

### Gut microbiome analyses

The data for each sample was split from the downstream data based on the barcode and PCR amplification primer sequences and after truncating the barcode and primer sequences, the reads for each sample were spliced using FLASH (V1.2.7, http://ccb.jhu.edu/software/FLASH/) to obtain the raw tags. Quality filtering of raw tags was performed under specific filtering conditions to obtain high-quality clean tags according to the QIIME (V1.9.1, http://qiime.org/scripts/split_libraries_fastq.html) quality-controlled process. Then the tags were then compared with a reference database (Silva database, https://www.arb-silva.de/) using the UCHIME algorithm (http://www.drive5.com/usearch/manual/uchime_algo.html) to remove chimeric sequences and obtain effective tags. Operational taxonomic units (OTUs) were analyzed for clustering and species classification based on effective data using UPARSE software(Edgar, 2013). Sequences with ≥ 97% similarity were grouped into the same OTU and representative sequences from each OTU were annotated with taxonomic information based on the Mothur algorithm using the Silva database. The community composition of each sample was assessed at different taxonomic levels (phylum, order, order, family, and genus) and compared among groups of taxonomic levels. Alpha and beta diversity analyses were performed using the QIIME software (V1.9.1) and R software (V4.3.1). The alpha diversity of the samples was described using the observed species and Chao1 and ACE indices and *p*-values were calculated using Wilcoxon’s test. Beta diversity was assessed using an unweighted UniFrac distance matrix and visualized using principal coordinate analysis (PCoA) and non-metric multidimensional scaling (NMDS) plots; while differences in microbial community composition between the two groups were compared using ANOSIM analysis. We used a hierarchical clustering method, the Unweighted Pair-group Method with Arithmetic Means(UPGMA), which interprets distance matrices using average linkage via the QIIME software (version 1.9.1), for comparing the similarity of the gut microbiota in each group of samples. Linear discriminant analysis effect size (LEfSe) was used to identify key microbial taxa that differed significantly between the two groups(Segata et al., 2011) with an LDA threshold greater than 3.0 (NovoMagic Cloud Platform, https://magic.novogene.com/). To reveal potential differences in metabolism, a phylogenetic investigation of communities by reconstruction of unobserved state analysis (PICRUSt2) based on the MetaCyc database was used to predict the functional pathways in the microbiota(Douglas et al., 2020). The relative predicted abundance of the MetaCyc pathways was calculated by dividing the abundance of each pathway by the sum of the abundance of all pathways per sample.

### Construction and validation of diagnostic models

For clinical modeling, univariate logistic regression analysis combined with ROC curve analysis was used to screen out clinical variables with significant predictive value (*p* < 0.05, AUC ≥ 0.7). These variables were then included in the multivariate logistic regression analysis and those with *p* < 0.05 were further screened as independent risk factors for ACS and included in the final model. The “Forestplot” package in R was used to generate a forest map to show the Odds Ratio (OR), lower/upper 95% CI, and *p*-value. For microbiome modelling, we built a 10-fold cross-validated random forest model via “randomForest” R package to identify the microbiota biomarkers. Further, three diagnostic models were developed: clinical, microbiome and combined models. The accuracy of each model was assessed using the AUC value for the area under the ROC curve. The internal validation of the models was carried out using a bootstrap resampling method with a total of 1,000 resamples and was implemented using the “caret” R package and the ROC curves were plotted using the “pROC” R package. Based on the selected clinical variables and gut microbiome, a nomogram was constructed using the R package “rms” and a visualised dynamic nomogram web page with an interactive interface was developed using the R package “DynNom” to facilitate clinical application. The calibration of the model was assessed by Hosmer-Lemeshow test and calibration curves using the “rms” and “ResourceSelection” R packages. Decision curve analysis (DCA) was also performed using the “rmda” R package to assess the clinical utility of the diagnostic model.

### Statistical analysis

The continuous variables were expressed as mean ± standard deviation or median and interquartile range (IQR), and categorical variables were expressed as frequencies (percentages). Analysis of differences between groups that conformed to normal distribution was performed using the independent samples t-test and non-normally distributed differences were compared using the Mann–Whitney test. Categorical variables between the two groups were analyzed using the chi-squared test. Correlations between microbiota and clinical parameters as well as metabolic pathways were analyzed using Spearman’s correlation coefficients and presented visually by the R package “pheatmap”. All data analyses were performed using SPSS software (version 26.0), GraphPad Prism 9.0, and R 4.3.1 software. *P* < 0.05 was considered statistically significant.

## Results

### Baseline characteristics of the participants

After rigorous screening and exclusion criteria, 66 individuals, including 29 patients with ACS and 37 healthy controls, were included in the study. As shown in **Table 1**, patients with ACS had significantly higher levels of white blood cells (WBC), neutrophils (NEU), aspartate aminotransferase (AST), alanine aminotransferase (ALT), creatinine (Cr), and uric acid (UA), as well as higher rates of smoking history and hypertension, and significantly lower levels of beats per minute (BPM) and left ventricular ejection fraction (LVEF) compared to healthy controls. No significant differences were observed in demographic data, including age, body mass index (BMI), history of drinking, diabetes mellitus, hyperlipidemia, systolic blood pressure (SBP), total bilirubin (TBIL), blood urea nitrogen (BUN), fasting blood glucose (FBG), and serum lipid levels between the two groups.

**Table 1:** Baseline characteristics of the participants.

Variables	ACS(n=29)	Control(n=37)	*P-*values
Age, years	57.17 ± 9.86	57.78 ± 12.9	0.833
Male, n (%)	26(89.66)	22(59.46)	0.006
BMI, kg/m^2^	24.97 ± 3.60	23.91 ± 3.24	0.213
Smoking, n (%)	14(48.28)	6(16.22)	0.005
Drinking, n (%)	4(13.79)	3(8.11)	0.157
Hypertension, n (%)	20(68.97)	10(27.03)	0.001
Hyperlipidemia, n (%)	4(13.79)	5(13.51)	0.974
DM, n (%)	3(10.34)	3(8.11)	0.754
Type of ACS			
STEMI, n (%)	7(24.14)	NA	NA
NSTEMI, n (%)	12(41.38)	NA	NA
UA, n (%)	10(34.48)	NA	NA
No. of stenosed vessels			
1, n (%)	11(37.93)	NA	NA
2, n (%)	9(31.03)	NA	NA
3, n (%)	9(31.03)	NA	NA
Gensini score	68.55 ± 32.07	NA	NA
SBP, mmHg	126.59 ± 17.57	121 ± 17.48	0.203
BPM	78.66 ± 11.47	87.78 ± 11.58	0.002
LVEF(%)	66.59 ± 7.06	71.41 ± 5.21	0.002
Laboratory results			
WBC, ×10^9^/L	7.99(6.04,10.72)	5.54(4.73,6.42)	<0.001
NEU, ×10^9^/L	5.64(3.42,8.35)	2.77(2.47,4.00)	<0.001
LYM, ×10^9^/L	1.61(1.14,2.25)	1.79(1.30,2.12)	0.752
Hb, g/L	151.28 ± 16.22	144.30 ± 17.41	0.101
PLT, ×10^9^/L	226.34 ± 56.98	234.46 ± 53.88	0.556
ALB, g/L	42.17 ± 6.58	41.26 ± 3.66	0.482
AST, U/L	36(18.15,67.55)	18.2(15.1,23.55)	<0.001
ALT, U/L	28.5(18.15,42.5)	18.6(13.75,25.55)	0.010
TBIL, umol/L	12.09 ± 3.85	10.75 ± 4.28	0.194
BUN, mmol/L	5.42 ± 1.81	5.78 ± 1.52	0.378
Cr, umol/L	86.46 ± 19.62	74.4 ± 13.66	0.005
UA, umol/L	411.98 ± 108.36	358.98 ± 93.68	0.037
FBG, mmol/L	6.34 ± 2.63	5.24 ± 1.87	0.063
TC, mmol/L	4.63 ± 1.11	4.64 ± 0.85	0.949
TG, mmol/L	1.88 ± 1.27	1.53 ± 0.83	0.182
LDL-C, mmol/L	2.77 ± 0.89	2.79 ± 0.72	0.928
HDL-C, mmol/L	1.09 ± 0.27	1.22 ± 0.30	0.073

Continuous variables are presented as mean ± SD or median (interquartile range). Categorical variables are expressed as n (%). BMI, body mass index; DM, diabetes mellitus; STEMI, ST-segment elevation myocardial infarction; NSTEMI, non-ST-segment elevation myocardial infarction; UA, unstable angina; LVEF, left ventricular ejection fraction; SBP, systolic blood pressure; DBP, diastolic blood pressure; BPM, beat per minute; WBC, white blood cells; NEU, neutrophil; LYM, lymphocyte; Hb, hemoglobin; PLT, platelets; ALB, albumin; AST, aspartate aminotransferase; ALT, alanine aminotransferase; TBIL, total bilirubin; BUN, blood urea nitrogen; Cr, creatinine; UA, uric acid; FBG, fasting blood glucose; TC, total cholesterol; TG, triglyceride; LDL-C, low-density lipoprotein cholesterol; HDL-C, high-density lipoprotein cholesterol.

### Data quality assessment and gut microbiota diversity

**Figure 1:**
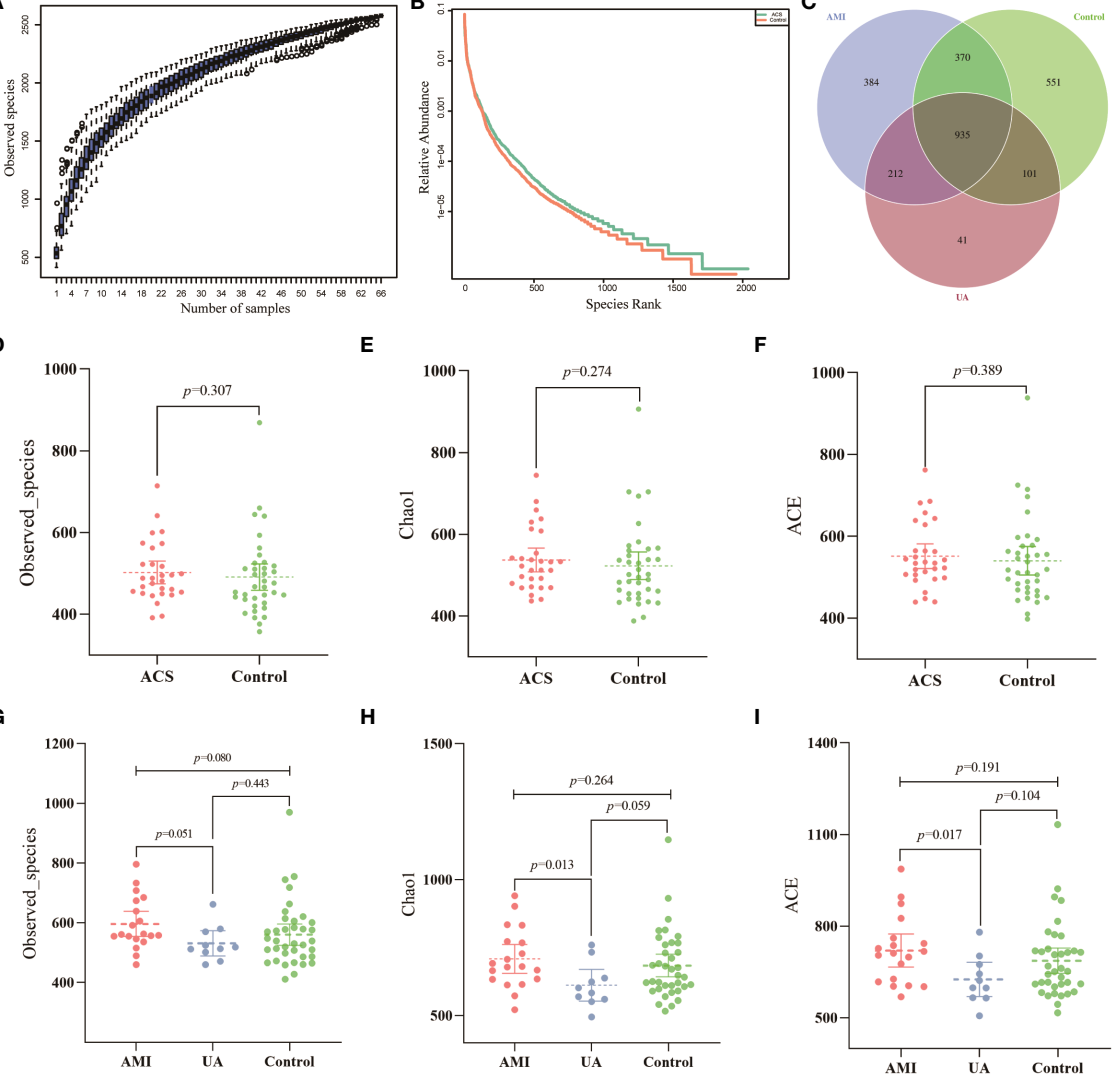
Data quality and alpha diversity of microbial sequences. **(A)** The species accumulation curve showed a flattening of the curve as the sample size increased, suggesting that the sample size was sufficient to show the richness of the community. **(B)** The rank abundance curve indicated high species diversity and good species evenness in the sample. **(C)** Venn diagram showing the number of unique OTUs and their shared OTUs in the AMI, UA and control groups. **(D)** Observed species index for ACS and control groups. **(E)** Chao1 index for ACS and control groups. **(F)** ACE index for ACS and control groups. **(G)** Observed species index for the three groups. **(H)** Chao1 index for the three groups. **(I)** ACE index for the three groups.

**Figure 2:**
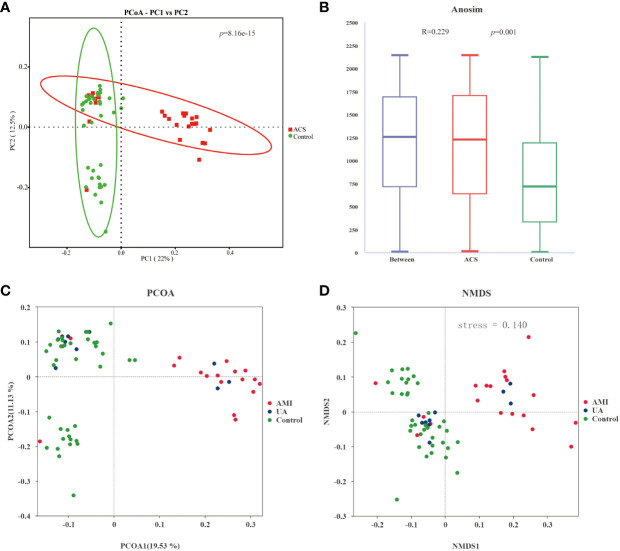
Analysis of the beta diversity of the gut microbiota. **(A)** PCoA score plot showing that samples from the ACS group (red) and the control group (green) were significantly separated (*p*<0.001). **(B)** ANOSIM showed a significant difference between the two groups (R = 0.229, *p* = 0.001). **(C)** PCoA score plot for AMI, UA and control groups. **(D) **NMDS analysis of AMI, UA and control groups(stress=0.14 (< 0.2)).

Gut microbiota analyses were performed using 16S rRNA sequencing of fecal samples from the included study population. To determine whether the sample size was sufficient to estimate the abundance of the microbial community, the species accumulation boxplot showed a gradual increase in species diversity with increasing sample size, with the curve flattening out at 66 samples (**Figure 1A**). This suggests that the current sequencing sample size was sufficient to detect most species in each sample. The abundance rank curves indicated high species richness and evenness in each sample group (**Figure 1B**). Through 16S rRNA gene sequencing, the sequenced samples were clustered into OTUs at a 97% similarity level, and 2614 OTUs were obtained. The Venn diagram (**Figure 1C**) displays the identified OTUs and shows a decreasing trend in the number of OTUs in patients with acute myocardial infarction (AMI) and unstable angina pectoris (UA) compared to the control group. In addition, the number of OTUs was significantly higher in the AMI group than in the UA group. Alpha-diversity analyses consistently showed similar results. Although no significant difference was observed in α-diversity between the ACS and control groups (**Figures 1D–F**), further subgroup analyses showed that the bacterial community richness and diversity were significantly increased in the AMI group compared to those in UA group (**Figures 1G–I**). To assess the overall structure of the gut microbiota, a principal coordinate analysis (PCoA) score plot was constructed based on the unweighted UniFrac distance. The results showed that the ACS group and healthy control group were separated, and the distribution between the two groups was approximately symmetrical (**Figure 2A**, *P* < 0.001). Analysis of non-parametric similarity (ANOSIM) further showed that the distribution and composition of the gut microbiota were significantly different between the two groups (R = 0.229, *p* = 0.001, **Figure 2B**). In addition, subgroup analyses using principal coordinate analysis (PCoA) score plots and non-metric multidimensional scaling (NMDS) analyses showed a clear separation between the AMI and control groups, with significant differences in the distribution of bacterial communities (**Figures 2C, D**).

### Composition and comparison of the gut microbiota in patients with ACS and healthy controls

**Figure 3:**
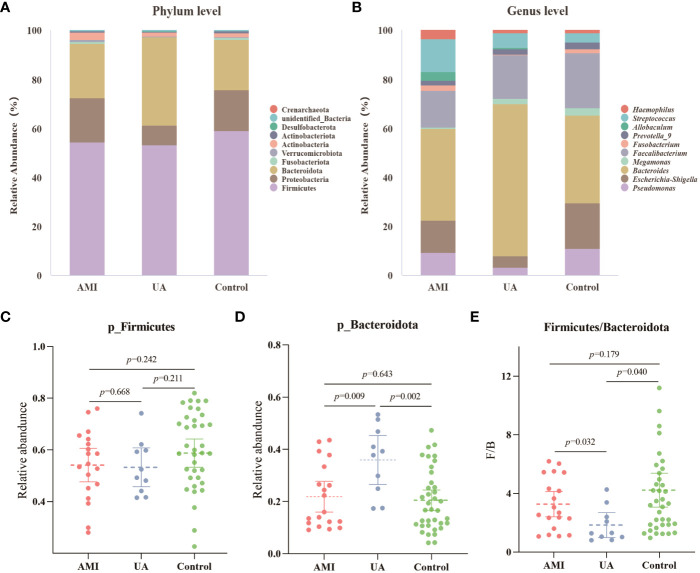
Analysis of microbial composition. **(A)** Composition of gut microbiota at the phylum level. **(B)** Composition of gut microbiota at the genus level. **(C)** Differences in abundance of *Firmicutes* phyla between the three groups. **(D)** Differences in abundance of *Bacteroidota* phyla between the three groups. **(E)** Differences in the ratio of *Firmicutes* to *Bacteroidota* (F/B) between the three groups.

**Figure 4:**
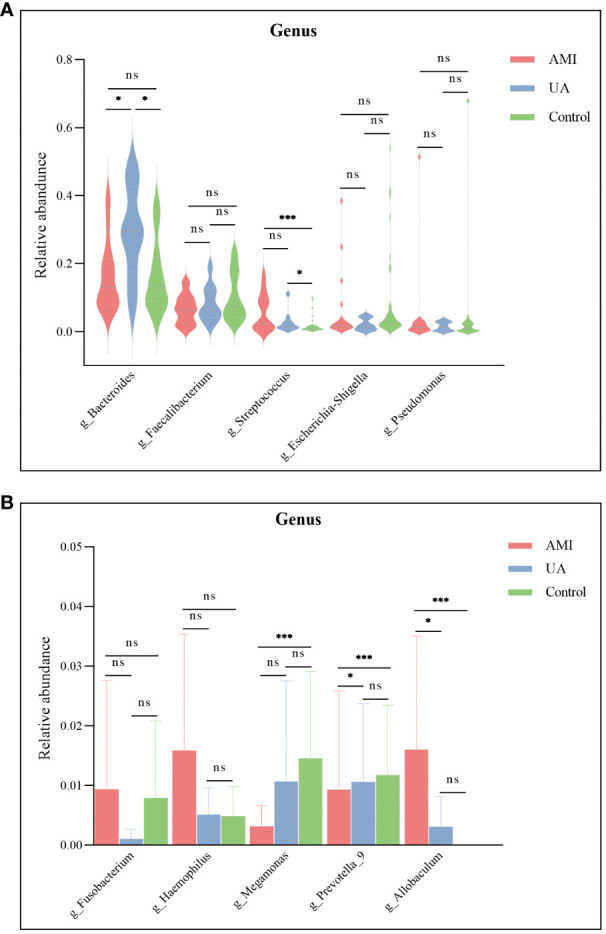
Differences in the relative abundance of major genera among the three groups. **(A)** Significant differences were observed in the genera *Bacteroides* and *Streptococcus*. **(B)** *Megamonas*, *Prevotella_9*, and *Allobaculum* were significantly different between the three groups. ns, no significance; *p < 0.05; **p < 0.01; ***p < 0.001.

**Figure 5:**
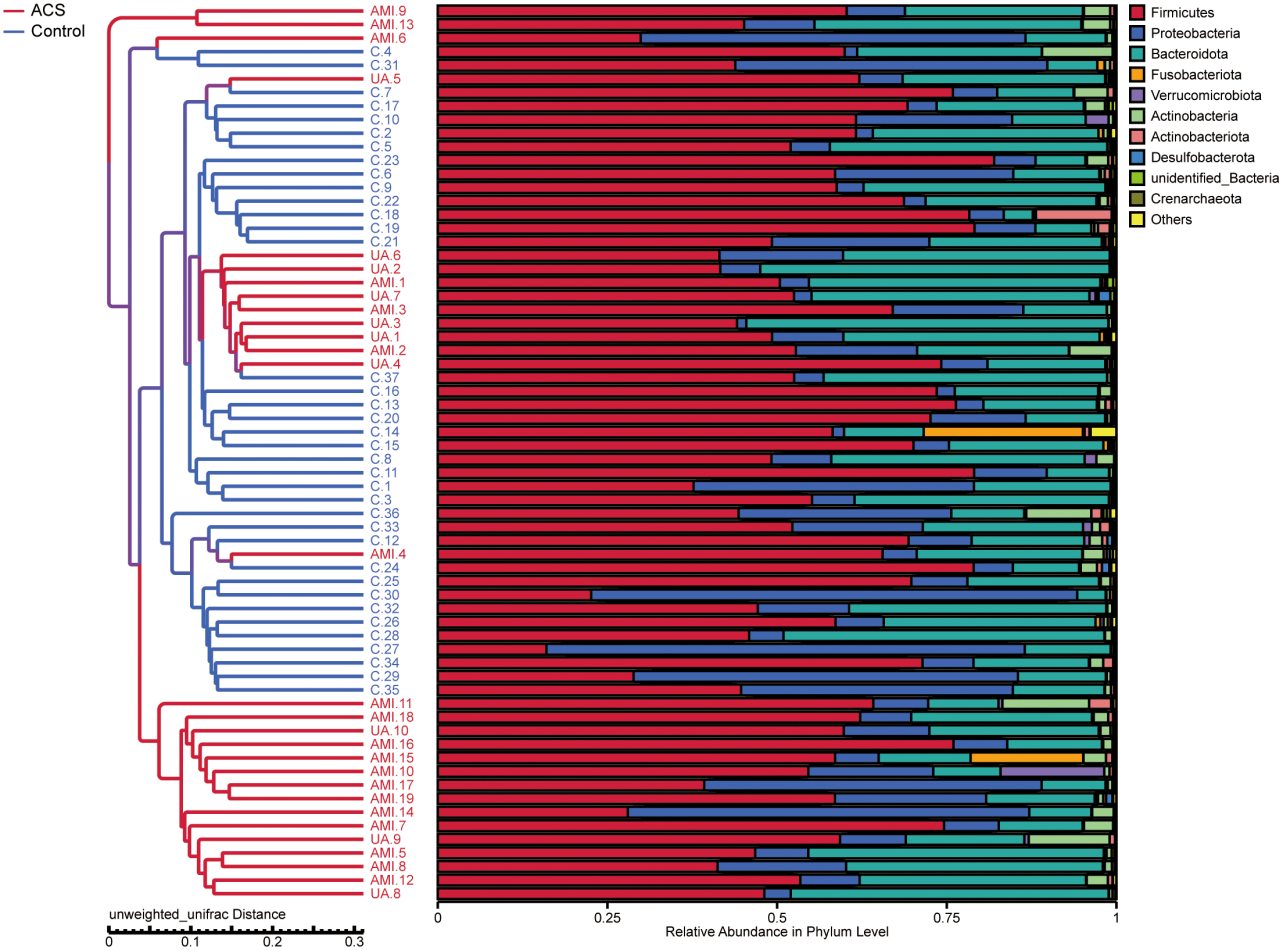
UPGMA (unweighted pair-group method with arithmetic mean) sample clustering tree showing the distribution of samples in the ACS and control groups.

**Figure 6:**
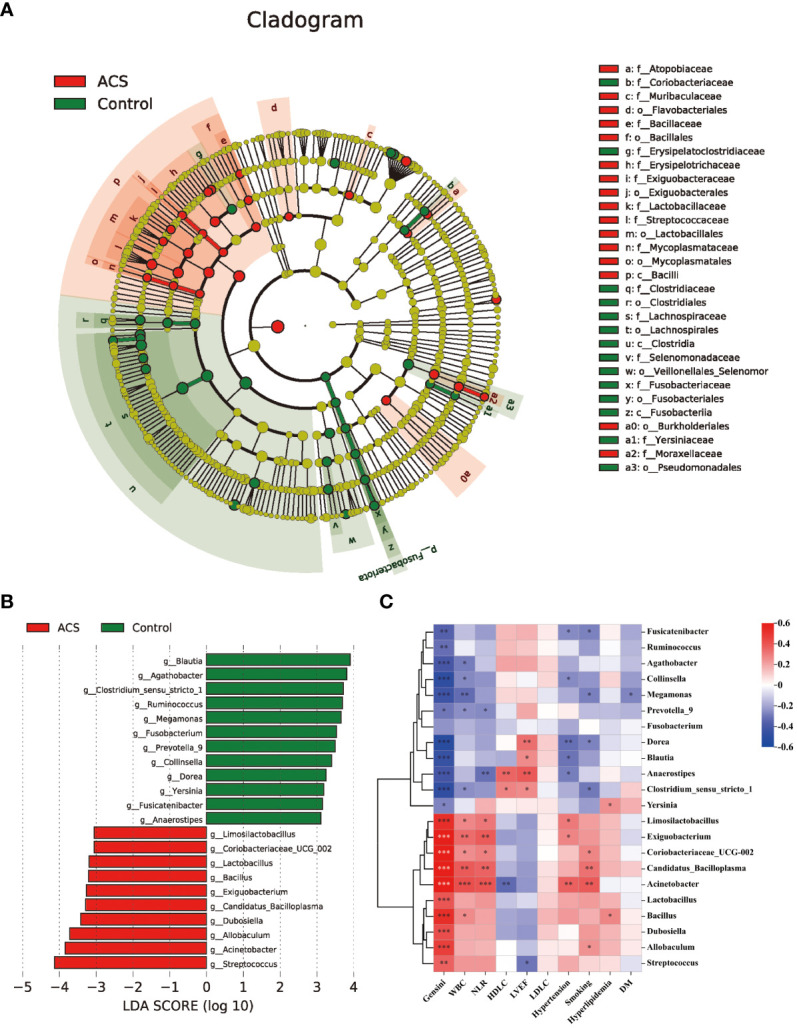
Analysis of specific differential microbiota in the ACS and control groups. **(A)** Cladogram generated by the LEfSe method showed the phylogenetic distribution of the gut microbiota associated with patients with ACS and healthy controls. **(B)** Histogram of LDA scores of the gut microbiota showing significant differences at the genus level between the ACS (red) and the control group (green). The default criteria LDA > 3 and *p* < 0.05 indicate that species are different, with one group being more abundant than the other. **(C)** Heat map of Spearman’s correlation between the differential genera and clinical characteristics. Colours represent positive (red) or negative (blue) correlations and *p* values are denoted as follows: *p < 0.05, **p < 0.01, ***p < 0.001. LVEF, left ventricular ejection fraction; WBC, white blood cell; NLR, neutrophils/lymphocytes.

Based on the species annotation results, the top ten species of phyla and genera with the highest relative abundance were selected, and relative abundance histograms were generated. At the phylum level, *Firmicutes* (AMI: 54.1%, UA: 53.3%, control: 58.7%) and *Bacteroidota* (AMI: 21.9%, UA: 35.9%, control: 20.5%) were the dominant phyla in all three groups (**Figure 3A**). The ratio of *Firmicutes* to *Bacteroidota* (F/B ratio) has been reported to be associated with metabolic disease (Magne et al., 2020). In our study, no significant differences were observed among the three groups in terms of the *Firmicutes* phylum (**Figure 3C**). The abundance of *Bacteroidota* was significantly higher, and the F/B ratio was significantly lower in the UA group than in the control and AMI groups (**Figures 3D, E**), which is consistent with the results of a previous multicenter study(Zheng et al., 2020). At the genus level, *Bacteroides* (AMI: 16.6%, UA: 29.2%, control: 16.3%) and *Faecalibacterium* (AMI: 6.6%, UA: 8.3%, control: 10.2%) were the most abundant genera in the three groups (**Figure 3B**). Further, we compared the differences in expression abundance among the three groups at the genus level and found that five genera were significantly different (**Figures 4A, B**). Overall, the genera *Bacteroides*, *Streptococcus* and *Allobaculum* were significantly more abundant in the case group than in the control group, whereas *Megamonas* and *Prevotella_9* were significantly less abundant. In addition, we observed subtle differences in the characteristics between the AMI and UA groups. In the UA group, a significant increase was observed in the genera *Bacteroides*, whereas the AMI group was characterized by a significant increase in *Streptococcus* spp. and *Allobaculum* spp abundance. To investigate the similarities between different samples, we constructed a cluster tree of the samples by UPGMA (Unweighted Pair-group Method with Arithmetic Mean) cluster analysis (**Figure 5**). The results showed that the clustering of the samples in the ACS and healthy control groups was clearly separated, whereas the AMI and UA samples in the ACS group were very close to each other, suggesting that the overall structure of the gut microbiota within the ACS group was similar. Therefore, we took acute coronary syndrome as a whole and further analysed the differences in gut microbiota between the ACS and control group, using linear discriminant analysis effect size (LEfSe) to identify specific differential genera between the two groups. The cladogram shows the phylogenetic distribution of the gut microbiota in patients with ACS and healthy controls (**Figure 6A**), and the LDA score plot showed that 22 genera were significantly different between the two groups (**Figure 6B**). Specifically, 10 genera such as *Streptococcus, Acinetobacter, Allobaculum and Dubosiella* were significantly enriched in ACS group; whereas 12 genera of *Blautia, Agathobacter, Clostridium_sensu_stricto_1, Ruminococcus* and *Megamonas* were more abundant in healthy controls (all *p*s < 0.05, LDA > 3).

### Correlations between the gut microbiome and clinical characteristics

Subsequently, we analyzed the correlation between these different genera and key clinical indicators to identify the key bacteria that are closely associated with the occurrence of ACS (**Figure 6C**). The results showed that the microbiota was significantly more strongly correlated with smoking, hypertension history, inflammation levels, and Genisi scores; whereas it was weakly correlated with lipid levels and a history of diabetes and hyperlipidemia. Specifically, we found that the microbiota was significantly associated with cardiovascular risk factors including smoking, hypertension, and levels of inflammation assessed by WBC count and NLR, with *Acinetobacter* showing the strongest positive correlations with WBC count (r = 0.419, *p* < 0.05) and NLR (r = 0.401, *p* < 0.05). In addition, the microbiota were strongly associated with cardiovascular protective factors, including HDL-C and LVEF, with *Anaerostipes* showing the highest positive correlation with HDL-C (r = 0.356, *p* < 0.05) and LVEF (r= 0.393, *p* < 0.05). Finally, we also observed that among these 22 genera, 21 genera had significant correlations with the severity of coronary atherosclerosis assessed by the Genisi score (10 positive and 11 negative), with *Acinetobacter* showing the highest positive correlation with the Genisi score (r= 0.799, *p* < 0.05) and *Dorea* showing the highest negative correlation with Genisi score (r= -0.511, *p*< 0.05). These results suggest that alterations in microbial communities, particularly those of *Acinetobacter*, *Dorea*, and *Anaerostipes*, may indicate changes in inflammation, metabolism, and severity of coronary atherosclerosis in patients with ACS.

### Microbial function prediction analysis

**Figure 7:**
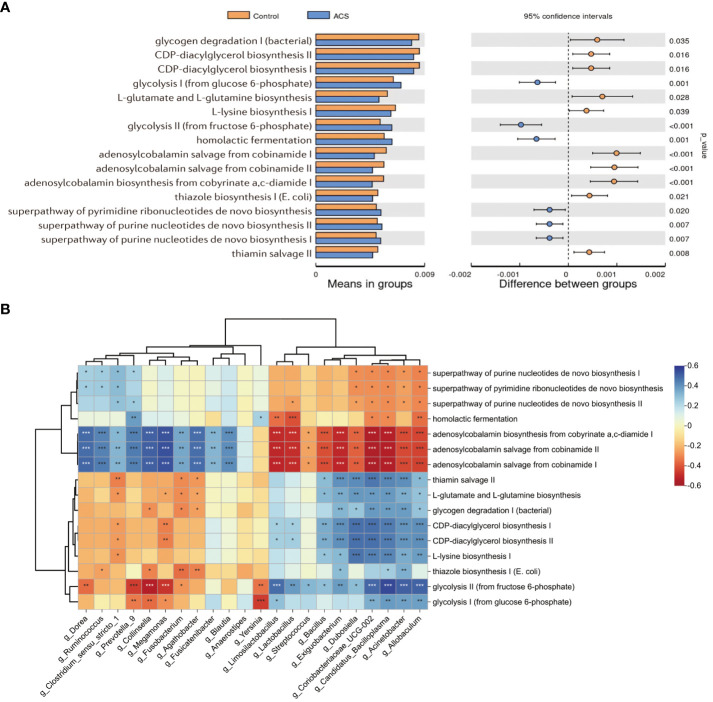
Microbial function prediction analysis. **(A)** PICRUSt2 analysis was used to predict alterations in metabolic pathways and showed that a total of 94 MetaCyc pathways were significantly different between the two groups, with a mean proportion of pathways greater than 0.005 being displayed. **(B)** The correlation heatmap demonstrated the association between major metabolic pathways and important differential genera. *p < 0.05, **p < 0.01, ***p < 0.001.

To investigate functional alterations in the microbial community of patients with ACS, we identified the functional potential of the gut microbiota using the PICRUSt2 tool based on the MetaCyc database. A total of 94 metabolic pathways were significantly different (*p* < 0.05, FDR < 0.2; **Figure 7A**, **Table S1**) (56 pathways enriched and 38 pathways depleted in the ACS group). The results showed that the pathways of glycolysis, homolactic fermentation, pyrimidine deoxyribonucleotides *de novo* biosynthesis, purine nucleotides *de novo* biosynthesis were enriched in the ACS group, whereas adenosylcobalamin biosynthesis, glycogen degradation, L-glutamate and L-glutamine biosynthesis, L-lysine biosynthesis and thiamin salvage were enriched in the control group. Notably, glycolytic metabolic pathways were highly enriched in the ACS microbiome, whereas adenosylcobalamin biosynthesis was significantly reduced, which may be related to the disease state. Furthermore, these metabolic pathways were closely associated with important differential microbiota (**Figure 7B**). The glycolytic pathway was positively associated with the ACS-enriched genera, particularly *Acinetobacter* and *Allobaculum*, whereas the adenosine synthesis pathway was positively associated with the ACS-negative genera.

### ACS diagnostic models based on gut microbiome and clinical features

**Figure 8:**
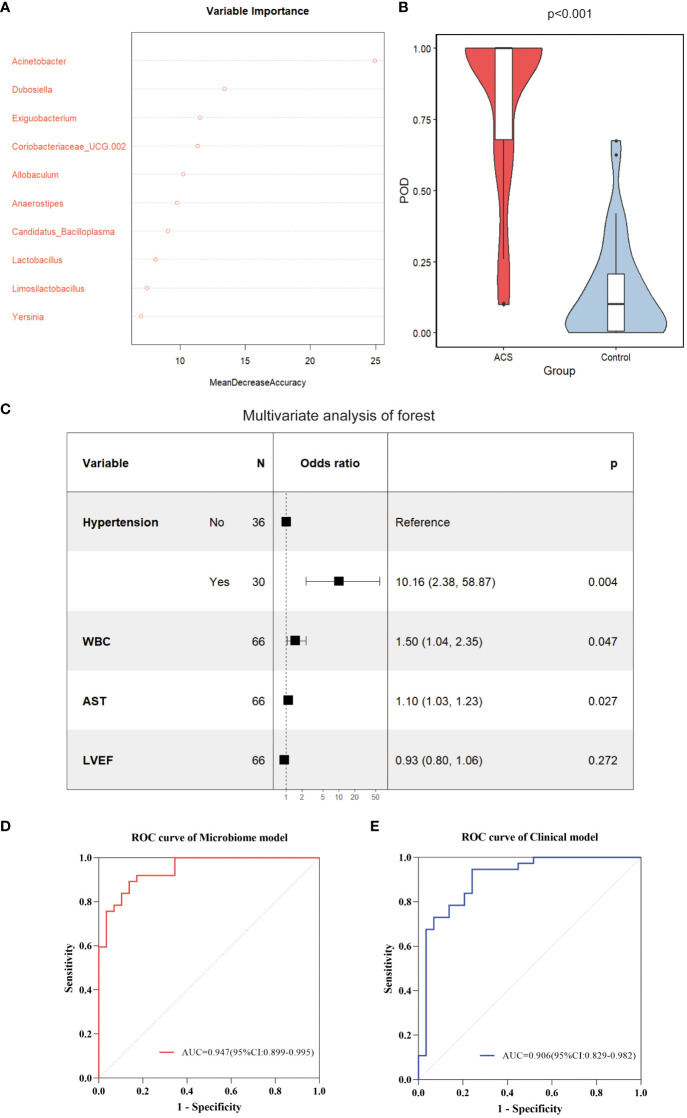
Identification of important microbial markers and clinical variables. **(A)** The top 10 genera most important for distinguishing ACS from healthy controls were screened by Random Forest (RF). Each genus was ranked according to mean decrease accuracy. **(B)** Comparison of gut microbiome POD index between ACS and control groups (*p* < 0.001). **(C)** Candidate variables for clinical model development were presented as forest plots. **(D)** ROC curves with AUC for diagnostic performance of the microbiome model. **(E)** ROC curves with AUC for diagnostic performance of the clinical model. POD, probability of disease; AUC, area under the curve.

Subsequently, to identify important microbial biomarkers for the construction of diagnostic models, we constructed a random forest model with a 10-fold cross-validation among the different genera screened. As shown in **Figure 8A**, random forest analysis filtered the top 10 genera that were most important for distinguishing patients with ACS from healthy controls based on the mean decrease accuracy index. Among the top five genera in terms of variable importance were selected as gut microbiome markers, including *Acinetobacter, Dubosiella, Exiguobacterium, Coriobacteriaceae_UCG.002*, and *Allobaculum*. Based on the five selected gut microbial markers, we calculated the Probability of Disease (POD) index, which reflects the diagnostic value of microbial markers in the ACS group and healthy controls(Zheng et al., 2020). As shown in **Figure 8B**, the POD index was significantly higher in ACS samples than in control samples (*p* < 0.001). The POD index was then used to construct the microbiome model and ROC curves were plotted, reaching an AUC value of 0.947 with a 95% CI of 0.899–0.995 (**Figure 8D**). These results indicated that the diagnostic model based on microbial markers had good diagnostic efficacy. Although the gut microbiome has performed well in diagnosing ACS, it alone may not be sufficient owing to the complexity of the disease. Therefore, we screened clinical indicators for inclusion in the diagnostic model to optimize their performance in disease prediction. To screen for candidate clinical variables, univariate regression and ROC curves (with AUC) were first utilized to screen for clinical indicators with *p* < 0.05 and AUC ≥ 0.7, and four predictive clinical factors were identified (**Table 2**). Multivariate logistic regression was then performed on these four factors, and the results showed that a history of hypertension (*p* = 0.004), elevated WBC count (*p* = 0.047), and elevated AST (*p* = 0.027) were independent risk factors for ACS (**Figure 8C**). Finally, these three independent risk factors were included in the logistic regression analysis to construct a clinical model with an AUC value of 0.906 (95% Cl: 0.829–0.982) (**Figure 8E**). This indicated that the diagnostic efficacy of the microbiome model was superior to that of the clinical model.

**Table 2:** Candidate variables for clinical model development.

Variables	AUC	*P-*values	95%CI
Male	0.651	0.011	0.043-0.662
BMI	0.562	0.214	0.947-1.276
Smoking	0.660	0.007	0.066-0.647
Hypertension	0.710	0.001	0.057-0.486
WBC	0.771	0.001	1.233-2.147
AST	0.778	0.003	1.030-1.151
ALT	0.687	0.331	0.990-1.029
BUN	0.417	0.374	0.642-1.181
Cr	0.686	0.010	1.011-1.085
UA	0.646	0.042	1.000-1.011
FBG	0.676	0.076	0.975-1.661
TC	0.506	0.948	0.591-1.636
TG	0.582	0.191	0.848-2.280
HDL-C	0.635	0.078	0.032-1.203
LDL-C	0.497	0.926	0.523-1.803
LVEF	0.709	0.005	0.798-0.961

### Combined model and nomogram for predicting ACS

**Figure 9:**
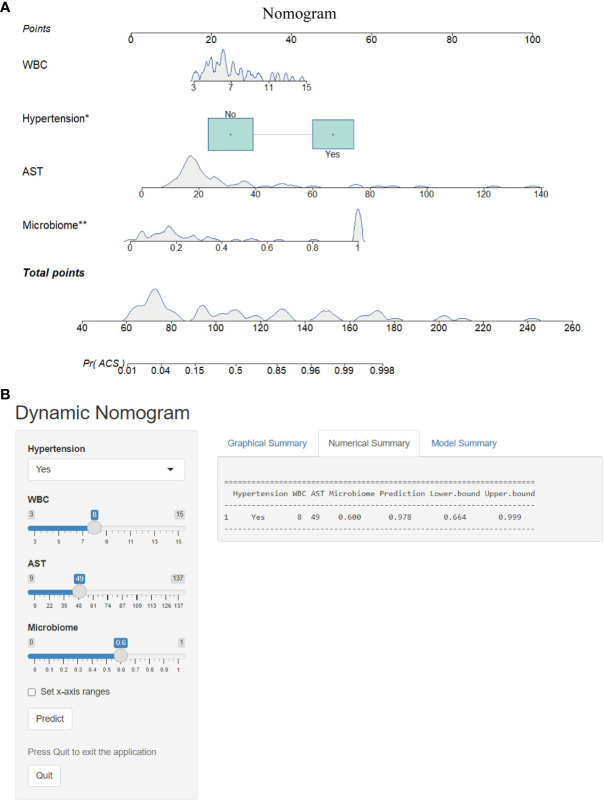
Nomogram and its webpage development. **(A)** The nomogram was constructed based on hypertension, WBC, AST and microbiome to predict the probability of developing ACS. To use the nomogram, a vertical line is drawn from the risk factor to the “Points” axis to determine the score for each risk factor in the nomogram. The scores for all risk factors are summed and a vertical line is drawn from the “Total Score” axis to the “Probability of ACS” axis, the corresponding value of which is the probability of developing ACS. **(B)** Web-based risk calculator (Dynamic Nomogram (https://wjcww.shinyapps.io/dynnomapp/) to predict incidence rate of ACS. *p < 0.05, **p < 0.01.

**Figure 10:**
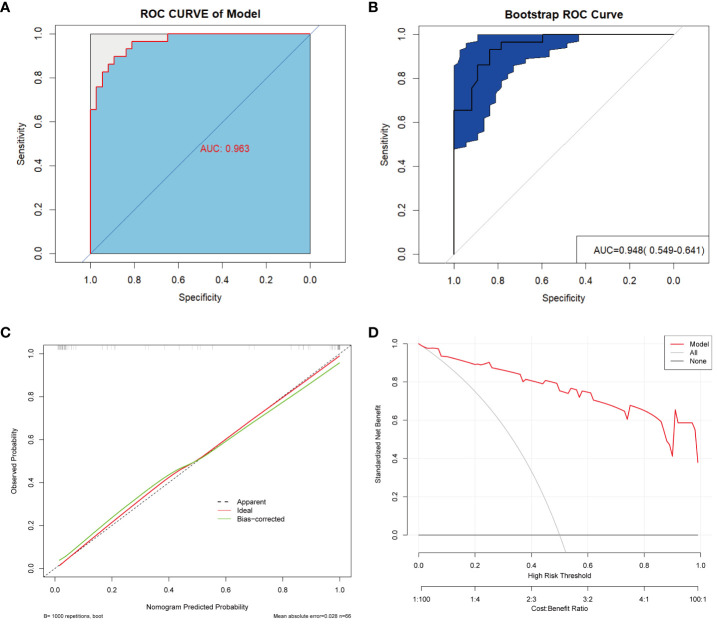
Validation and assessment of the model. **(A)** ROC curve with AUC for the diagnostic performance of the Nomogram. **(B)** The AUC for Nomogram bootstrap internal validation. **(C)** Calibration curve of the nomogram, with the x-axis representing the probability predicted by the nomogram and the y-axis representing the actual observed probability. **(D)** Decision curve analysis (DCA) of the nomogram showed the net benefit of using the model to diagnose ACS compared to the “treat all” or “treat none” strategy at different decision thresholds.

To optimize diagnostic efficiency, we constructed a combined diagnostic model by combining the gut microbiome POD index with screened clinical indicators and developed a nomogram to visualize the risk of ACS (**Figure 9A**). Simultaneously, a web-based dynamic nomogram was developed to predict the risk of ACS and to its facilitate clinical application (https://wjcww.shinyapps.io/dynnomapp/). For example, patients were randomly selected from a population. The patient was diagnosed with hypertension, with a WBC of 8 x10^9^/L, and an AST level of 49 U/L. Microbiological tests were performed on the stool samples, and the POD index was calculated as 0.6. Entering the above information into this diagnostic model, the probability of ACS in this patient is 97.8%, and the results are shown in **Figure 9B**. The results showed a higher predictive power of the combined model than that of the clinical model (AUC: 0.963 vs. AUC: 0.906) or microbiome model (AUC: 0.963 vs. AUC: 0.947) alone(**Figure 10A**). In addition, the consistency index (C-index) of 0.951 was used to assess the diagnostic performance of the combined mode. For internal validation, we used the bootstrap method to internally validate the model with 1,000 bootstrap resamples, resulting in an AUC value of 0.948, sensitivity of 0.89, and specificity of 0.83 (**Figure 10B**). The model showed good diagnostic efficacy during resampling, indicating that it was stable. Regarding the assessment of the model calibration, a Hosmer-Lemeshow goodness-of-fit test was performed, which resulted in *p* = 0.729 (>0.05), and the calibration curves also showed no significant deviation between the observed and predicted probabilities (**Figure 10C**). To assess the utility of the model in decision-making, a decision curve analysis was performed. As shown in **Figure 10D**, the model curve deviated from the two extreme curves (none and all), indicating that the diagnostic model yielded a high net clinical benefit in patients with ACS. These results suggest that a diagnostic model based on the gut microbiome and clinical variables has favorable diagnostic efficacy and utility.

## Discussion

Recent studies have found that the gut microbiota can be used as noninvasive biomarkers for the early diagnosis and prognostic assessment of disease; however, differences in the gut microbiota and the efficiency of the models are affected by regional variations(He et al., 2018). Therefore, investigating the characteristics of the gut microbiota in patients from different regions is crucial for elucidating possible pathogenic mechanisms and establishing diagnostic models. In this study, we first characterized the differences in gut microbiota between patients with ACS and healthy people in Southwest China and constructed a diagnostic model based on the microbiome, which significantly improved the diagnostic accuracy in patients with ACS and had a high clinical utility value.

A balanced intestinal microecology is important for maintaining normal physiological functions in the human body(Xu et al., 2020). Our study showed that the diversity and composition of the gut microbiota were significantly disturbed in patients with ACS compared to those in healthy controls. LEfSe analysis revealed that certain potentially pathogenic bacteria were significantly enriched in the ACS group, such as *Streptococcus* spp. and *Acinetobacter* spp. This is not the first time that the relationship between *Streptococcus* spp. and coronary artery disease has been reported. More than a decade ago, Koren et al.(Koren et al., 2011)detected *Streptococcus* spp. in the gut and oral cavity of patients with atherosclerosis and the same DNA was detected in atherosclerotic plaque samples. Recent studies have shown that the abundance of *Streptococcus* spp. is significantly increased in patients with subclinical atherosclerosis(Sayols-Baixeras et al., 2023), stable coronary artery disease(Jie et al., 2017), and acute myocardial infarction(Dong et al., 2023), and has a high predictive value for disease. Our study found that the abundance of *Streptococcus* spp. was significantly elevated in both the AMI and UA groups compared to the control group, however, this elevation was more pronounced in the AMI group (**Figure 4A**, *p*<0.001). This indicates that changes in *Streptococcus* spp. abundance may be associated with the formation of atherosclerotic plaques or thrombi and may be a valuable marker for detecting the progression of ACS. Furthermore, we found that the number of OTUs and the diversity of the gut microbiota were also significantly higher in the AMI group than in the UA group, which is different from the results of other studies(Gao et al., 2020a; Qian et al., 2022). This may be due to the unique pathophysiological processes of acute myocardial infarction, such as acute thrombosis, myocardial necrosis, inflammation, and activation of the neuroendocrine system, which may cause changes in the characteristics of the gut microbiota in patients with AMI. Ventricular dysfunction after acute myocardial infarction may lead to hemodynamic disturbances, such as inadequate intestinal perfusion and congestion, which can lead to increased intestinal permeability and intestinal dysfunction, allowing the intestinal microbiota to divert into circulation and cause endotoxemia, which may exacerbate the onset and progression of AMI(Zhou et al., 2018). Therefore, further study of the mechanisms of gut microbiota translocation may contribute to improving the diagnosis and treatment of myocardial infarction.

Notably, we found that abnormal enrichment of *Acinetobacter* spp. seemed to have a significant impact on the diagnosis of ACS. A small cohort study found that *Acinetobacter* was the most commonly detected genus in the coronary balloons of patients with obstructive coronary atherosclerosis(Serra e Silva Filho et al., 2014). In addition, a recent prospective cohort study found that *Acinetobacter* was also detected in cerebral thrombus samples from patients with large-vessel occlusive stroke, and that the abundance of *Acinetobacter* was positively associated with the risk of perioperative adverse events and death within three months(Liao et al., 2022). Therefore, we hypothesized that *Acinetobacter* in the intestine may be transferred to coronary atherosclerotic plaques or thrombi via blood circulation, thereby exacerbating the formation of inflammation and the progression of atherosclerotic plaques. However, this requires further study. However, we did not perform 16S rRNA gene sequencing of coronary plaque or thrombus samples, which is a limitation of our study. Although the relationship between *Acinetobacter* and ACS remains unclear, we observed that the abundance of *Acinetobacter* in the gut was significantly and positively correlated with the level of inflammation and the severity of coronary atherosclerosis. In addition, microbial function prediction analyses have shown a significant positive correlation between *Acinetobacter* and the glycolytic pathway, which is the most critical pathway for glucose metabolism in humans(Chen et al., 2023). Studies have found that during myocardial ischemia-reperfusion, myocardial metabolism shifts from oxidative phosphorylation to aerobic glycolysis, leading to an abnormal accumulation of glycolytic intermediates. This drives mitochondrial dysfunction and increases the formation of reactive oxygen species (ROS), further leading to the apoptosis of cardiomyocytes(Ait-Aissa et al., 2019; Dambrova et al., 2021). Therefore, further investigation of the link and mechanism between *Acinetobacter*, glycolytic metabolic pathways, and ACS is of great interest, and will provide potential opportunities for microbial metabolic pathways as targets for therapeutic intervention in cardiovascular disease.

Contrarily, the genera *Blautia, Agathobacter, Ruminococcus, Dorea*, and *Anaerostipes* were depleted in patients with ACS and significantly enriched in healthy controls. These genera have been reported to ferment carbohydrates to produce short-chain fatty acids, which are essential for maintaining the integrity of intestinal epithelial cells and preventing bacterial translocation into the bloodstream and subsequent endotoxaemia(Makki et al., 2018; Wan et al., 2019). In our study, the genus *Blautia* had the highest LDA values among the healthy controls and was negatively correlated with the severity of coronary atherosclerosis. Gao et al. (Gao et al., 2020b) showed that *Blautia* may play an important role in α-linolenic acid-mediated improvement in intestinal barrier integrity and anti-inflammatory effects, and that exacerbation of inflammation is critical in the pathophysiology of ACS (Dziedzic et al., 2022). In addition, a Mendelian randomization relating gut microbiota to ischaemic heart disease and its risk factors showed nominal associations of *Acidaminococcus*, *Aggregatibacter*, *Anaerostipes*, *Blautia*, *Desulfovibrio*, *Dorea*, and *Faecalibacterium* with a modestly lower risk of T2DM, lower adiposity, more beneficial lipid profiles, and higher HOMA-IR(Yang et al., 2018).

Several studies have shown that the gut microbiota and metabolic profiles can be altered through dietary interventions, which may have a significant impact on cardiovascular risk factors(So et al., 2018; Wan et al., 2019). For example, dietary intervention with high-fiber rye foods resulted in changes in the composition of the gut microbiota and increased the abundance of butyric acid-producing *Agrobacterium*, which may be associated with intervention-induced weight loss and improvement in metabolic risk indicators(Iversen et al., 2022). In addition, statins have been reported to modulate the gut microbiota of patients with ACS, increasing beneficial flora (such as *Bifidobacterium longum subsp. longum, Anaerostipes hadrus* and *Ruminococcus obeum*) to a healthier state, thus reducing the metabolic risk of patients(Hu et al., 2021). These results suggest that targeted modulation of gut microbiota through probiotic supplementation may be a novel approach for the prevention and treatment of cardiovascular diseases. However, there are few reports on the mechanisms by which probiotics improve cardiovascular disease, which should be a direction for future research.

In our study, we found that hypertension, WBC count, and AST levels were independent risk factors for ACS, consistent with the results of previous studies(Kaminska et al., 2018; Li et al., 2021). However, studies have found that up to 20% of patients with ACS do not have traditional clinical risk factors (Figtree and Vernon, 2021), limiting the clinical application of predictive models that include only clinical indicators. Therefore, we combined clinical variables with the gut microbiome to construct a combined diagnostic model with an AUC value of 0.963. The predictive power of the combined model was significantly better than that of the other two models. More importantly, even after bootstrap internal validation, the model showed good performance (AUC = 0.948), indicating that our model was stable.

The gut microbiota has been used to predict coronary artery disease in several recent studies and has shown high a predictive value(Zhang et al., 2022; Dong et al., 2023), suggesting the potential of the gut microbiota to predict ACS. However, these studies only constructed predictive models and did not validate the calibration and utility of the models, making it difficult to generalize the models for clinical use because of the lack of clinical application tools. Considering the importance of the early diagnosis of ACS, we developed a nomogram and a corresponding online webpage based on the gut microbiome and clinical indicators to visualize the model and assist in the clinical diagnosis and risk assessment of ACS. A previous study constructed a disease classifier based on a combination of 24 bacterial co-abundance groups (CAGs) and 72 serum metabolites, which accurately differentiated between stable coronary artery disease and acute coronary syndromes, with an AUC value of 0.897(Liu et al., 2019). However, this model requires the incorporation of many microbial indicators as well as invasive blood sampling to detect metabolites and is relatively complex and expensive to implement, which may limit its use in clinical settings. Conversely, our model was characterized by its simplicity, non-invasiveness, and accuracy, which was achieved by incorporating only a few microbiota and common clinical indicators. In our study, all participants were from the same region, and their lifestyles and diets were similar, which reduced the potential confounding effects of geographic and dietary differences on the microbiota. In addition, our study population included newly diagnosed and untreated patients with ACS who were at a relatively early stage of the disease, which reduced the impact of confounding factors, such as medication and disease progression, on the microbiome analysis. Therefore, the developed model is more informative for the early diagnosis of ACS.

Our study has several limitations. First, we only sequenced the 16S rRNA gene in fecal samples; we did not assess the metabolites of the microbiota, and the mechanisms are understudied. Second, although we established an accurate diagnostic model based on the gut microbiota, the specific functions of these microbiota remains unclear. Finally, this was a single-center study with a limited sample size, which did not allow for external validation in different regions. Bootstrap resampling was performed to ensure internal validity.

In conclusion, the current study showed that the diversity and composition of the intestinal mycobiota of patients with ACS was significantly disturbed and was characterized by significant enrichment of certain potentially pathogenic genera and a significant reduction in certain SCFA-producing genera. Our study provides novel insights into the association between the gut microbiota and ACS and more targeted studies of these critical microbiota will be valuable in the future. In addition, we constructed a noninvasive diagnostic model based on the gut microbiome and common clinical indicators, providing a novel approach to assist in the early diagnosis and risk warning of ACS.

## Data availability statement

The datasets presented in this study can be found in online repositories. The names of the repository/repositories and accession number(s) can be found below: https://www.ncbi.nlm.nih.gov/sra/PRJNA1020457.

## Ethics statement

The studies involving humans were approved by the Ethics Committee of the First Affiliated Hospital of Kunming Medical University. The studies were conducted in accordance with the local legislation and institutional requirements. The participants provided their written informed consent to participate in this study. Written informed consent was obtained from the individual(s) for the publication of any potentially identifiable images or data included in this article.

## Author contributions

JW: Visualization, Writing – original draft, Conceptualization, Data curation, Formal Analysis, Investigation, Methodology, Software. ZH: Conceptualization, Funding acquisition, Resources, Writing – review & editing, Supervision, Validation. QX: Conceptualization, Investigation, Methodology, Supervision, Writing – original draft. YS: Formal Analysis, Funding acquisition, Methodology, Resources, Software, Writing – original draft. XC: Resources, Data curation, Methodology, Writing – original draft. YM: Data curation, Investigation, Methodology, Writing – original draft. MW: Data curation, Investigation, Writing – original draft. CZ: Conceptualization, Investigation, Methodology, Writing – original draft. XL: Data curation, Methodology, Software, Writing – original draft. FL: Data curation, Investigation, Methodology, Writing – original draft. XBL: Data curation, Investigation, Methodology, Writing – original draft. YD: Conceptualization, Funding acquisition, Investigation, Project administration, Resources, Supervision, Validation, Writing – review & editing. HC: Conceptualization, Funding acquisition, Investigation, Project administration, Resources, Supervision, Writing – review & editing, Formal Analysis, Methodology, Validation, Visualization.
